# Phosphorylation of S‐S‐S Motif in Nuclear Export Protein (NEP) Plays a Critical Role in Viral Ribonucleoprotein (vRNP) Nuclear Export of Influenza A and B Viruses

**DOI:** 10.1002/advs.202309477

**Published:** 2024-11-22

**Authors:** Xiaokun Liu, Cha Yang, Xian Lin, Xiaomei Sun, Huanchun Chen, Qiang Zhang, Meilin Jin

**Affiliations:** ^1^ National Key Laboratory of Agricultural Microbiology Huazhong Agricultural University Wuhan 430070 P. R. China; ^2^ Wuhan institute of Virology Chinese academy of Science Wuhan 430070 P. R. China; ^3^ College of Veterinary Science and Medicine Huazhong Agricultural University Wuhan 430070 P. R. China; ^4^ Hubei Jiangxia Laboratory Wuhan 430200 P. R. China

**Keywords:** ATM, CK2, influenza virus, NEP S‐S‐S motif phosphorylation, vRNP nuclear export, vRNP nuclear export

## Abstract

The phosphorylation of three highly conserved serine residues S23, S24, and S25 (S‐S‐S motif) has been previously identified in NEP of influenza virus. However, it remains obscure whether and how this motif regulates the vRNPs nuclear export. Here the influenza A H5N6 viruses harboring NEP S23C, S24L, or S25L is generated, allowing to impair the phosphorylation on these sites without mutating viral NS1 protein. These mutations significantly inhibited vRNPs nuclear export are founded, decreased viral infectivity and attenuated virulence in mice. In addition, inhibition or knockout of ATM or CK2, two predicated Ser/Thr protein kinases that phosphorylate the S‐S‐S motif, impedes vRNP nuclear export and virus replication in cells and reduces the virulence in vivo. Moreover, treatment of NEP peptide mimics containing the S‐S‐S motif to competitively block NEP binding to the kinases reduces influenza virus replication in cells and mice. However, neither the inhibitors above nor the NEP peptide mimics significantly inhibit the replication of H5N6‐DDD mutant, indicating phosphorylation of S‐S‐S motif is required for the vRNP nuclear export. This studies contribute to a better understanding of the mechanism by which NEP regulates vRNP nuclear export and provides novel insights into antiviral targets against influenza A and B viruses.

## Graphical abstract

The phosphorylation of S‐S‐S motif on influenza virus NEP is required for NEP interaction with CRM1 thereby mediating vRNP nuclear export, which can be regulated by the kinase ATM and CK2. Impairment of phosphorylation on NEP S‐S‐S motif significantly suppresses virus replication and reduces the virulence in mice.

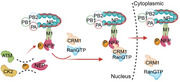

## 1 Introduction

The nuclear export of influenza viral ribonucleoprotein (vRNP), composed of genomic RNA, viral nucleoprotein (NP), and trimetric RNA‐dependent RNA polymerase complex, is an essential step for the viral life cycle, which is dependent on the nuclear export receptor chromosome region maintenance 1 (CRM1)‐mediated nuclear export pathway.^[^ ^1^, ^2^ ^]^ The viral nuclear export protein (NEP), also known as non‐structural protein 2 (NS2), plays a key role in the vRNP nuclear export.^[^ ^3^, ^4^ ^]^ Based on the current model for vRNP nuclear export, vRNPs are exported from the nucleus by forming a “daisy chain” complex, in which NEP acts as an adaptor bridging vRNP‐M1 (matrix protein 1) and CRM1‐GTPase Ran‐GTP.^[^ ^1^, ^5^, ^6^, ^7^ ^]^ Nevertheless, the detailed mechanism by which NEP regulates vRNP nuclear export remains to be fully dissected.

Three nuclear export signals (NES) have been identified in NEP of influenza A and B viruses, of which NES1 and NES2 are located at the N‐terminal and NES3 is in the C‐terminal domain of NEP.^[^ ^8^, ^9^, ^10^ ^]^ It is known that NES regulates NEP function in vRNP nuclear export. As demonstrated previously, NES1 and NES2 coordinately regulate the NEP‐CRM1 interaction,^[^ ^9^, ^11^ ^]^ while the exact roles of these two NESs during this process remain elusive. Interestingly, the phosphorylation of three highly conserved serine residues S23, S24, and S25 that resides between the NES1 and NES2 of NEP has been identified,^[^ ^12^, ^13^, ^14^ ^]^ however, its function is required to be further characterized.

In this study, we aim to investigate the role of phosphorylation on the S‐S‐S motif of NEP in influenza virus replication. We show a crucial role of the NEP S‐S‐S motif for viral replication of both influenza A and B viruses. Additionally, we reveal that the S‐S‐S motif is involved in regulating the function of NEP in influenza vRNP nuclear export. Our studies provide novel insights into the development of antiviral strategies or targets for influenza viruses.

## 2 Results

### 2.1 S23C, S24L, and S25L Mutations in NEP Reduce Influenza Viral Replication

**Figure 1:**
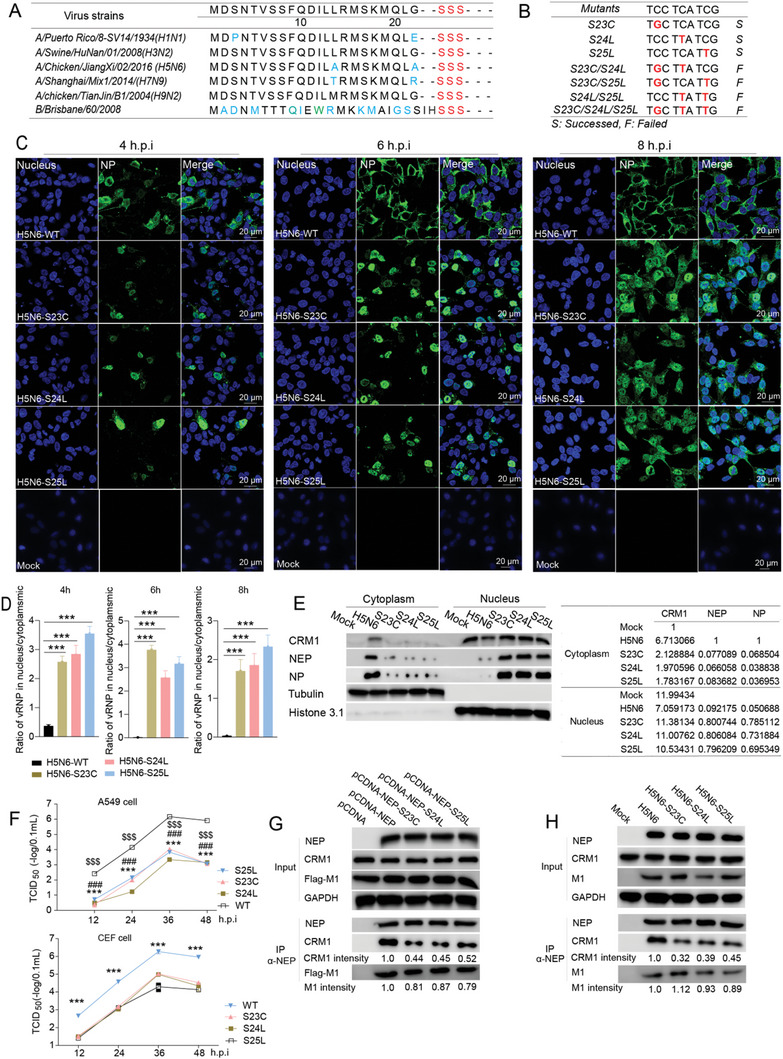
NEP S23C, S24L, and S25L mutations inhibit influenza A viral replication and vRNP nuclear export. A) Alignment of the NEP amino acid sequence from different influenza virus strains. S‐S‐S motif is found at the serine residues 23–25 and 25–27 in NEP of type A and B influenza virus, respectively. The S‐S‐S motif is highlighted in red. B) Nucleotide sequence design for the S23C, S24L, and S25L mutants of H5N6 influenza viral NEP. The mutations were highlighted in red. The NEP with the double‐ and triple‐mutations failed to be rescued. C and D) The effect of S23C, S24L, and S25L on influenza A vRNP nuclear export. A549 cells were infected with H5N6 wild‐type (WT) and the indicated NEP mutant virus, and NP distribution was detected at 4, 6, and 8 hours post‐infection (hpi.) (C). Representative images from three independent experiments were shown. Scale bar, 20 µm. The NP intensity in the nucleus and total NP intensity were quantified, and the ratio of intensity in the nucleus and cytoplasmic was shown (D). Data are presented as mean ± SD (*n* = 3), ***p < 0.001, two‐tailed Student's *t*‐test. E) The levels of NEP, NP, and CRM1 in cytoplasm and nucleus of the cells infected with H5N6 WT virus or NEP mutant virus. A549 cells were infected with the indicated viruses for 6 h, and the cytoplasmic and nuclear proteins were extracted followed by western blot analysis using the indicated antibodies. Tubulin and Histone 3.1 were used as cytoplasmic and nuclear markers, respectively, and CRM1, NEP, and NP levels in cytoplasm and nucleus were quantified and normalized to tubulin or histone 3. F) The effect of S23C, S24L, and S25L on influenza A virus replication. A549 or primary chicken embryo fibroblast (CEF) cells were infected with H5N6 WT viruses or the NEP mutant virus at an MOI of 0.01 for 12, 24, 36, and 48 h. The viral titer was then measured by TCID_50_ assay. Data are presented as mean ± SEM (*n* = 3). *** (S23C versus WT, for CEF cell group, * mutants versus WT), ^###^ (S24L versus WT), and ^$$$^ (S25L versus WT), *p* < 0.001, two‐tailed Student's *t*‐test. (G and H) The effects of S23C, S24L, and S25L mutations on NEP‐CRM1 and NEP‐M1 interactions. HEK293T cells were co‐transfected with p3xflag‐M1 and pcDNA‐NEP or the mutants and were lysed at 24 h post‐transfection, and Co‐IP was performed using an anti‐NEP antibody G). A549 cells were infected with H5N6 WT, NEP S23C, S24L, or S25L mutant viruses for 6 h and Co‐IP was performed using an anti‐NEP antibody H). CRM1 and M1 levels in IP were quantified.

Three conserved serine residues S23, S24, and S25, located between the NES1 and NES2, have been identified in the NEP of influenza A virus in previous study.^[^ ^13^, ^15^ ^]^ We further analyzed NEP sequences derived from the strains of different types of influenza viruses, the three serine (S) residues at positions 25, 26, and 27, were also found in the influenza B virus but not in the influenza C virus (**Figure** **1**A), suggesting that these serine residues, here referred to as S‐S‐S motif, are conserved among type A and B influenza viruses. Given the critical role of NEP in vRNP nuclear export, we hypothesized that the phosphorylation of the S‐S‐S motif might be involved in the NEP‐mediated nuclear export of vRNP, thereby regulating the influenza virus replication. To determine the effect of S‐S‐S phosphorylation on virus replication, reverse genetics was used to generate the influenza H5N6 mutant viruses harboring cysteine (C), leucine (L), and L at the position 23, 24, and 25 in NEP (Figure 1B), respectively, which enable to impair the phosphorylation of these sites without altering NS1 amino acid sequences. We found only the viruses with the single mutation were rescued, indicating that virus survival requires retention of at least two sites of the S‐S‐S motif (Figure 1B). The proliferation of the H5N6 wild‐type (WT) virus and NEP S23C, S24L, or S25L mutant viruses was then measured in the A549 cells and the primary chicken embryo fibroblast (CEF) cell. The results showed that the viral titers of mutant viruses were significantly lower than the WT virus, and no significant difference was found among the mutant viruses (Figure 1F), suggesting either S23, S24, or S25 in NEP are essential for efficient replication of influenza virus in both mammalian and avian cells.

### 2.2 S23C, S24L, and S25L Mutations in NEP Impair Influenza vRNP Nuclear Export

Next, the effect of the NEP S‐S‐S motif on the influenza vRNPs nuclear export was determined. A549 cells were infected with the WT H5N6 virus or the NEP mutant viruses, and the viral nucleoprotein NP was stained to visualize vRNP distribution in the cells. The data showed that the vRNPs were mainly distributed in the cytoplasm in the WT virus‐infected cells at 4 hours post‐infection (hpi) and completely localized in the cytoplasm after 6 hours of infection (Figure 1C,D). In contrast, the majority of the vRNPs were retained in the nucleus of the cells infected with the mutant viruses even at 8 hpi (Figure 1C,D), suggesting the vRNP nuclear export is largely delayed by NEP S23C, S24L, and S25L. Meanwhile, western blot analyses showed that the NP and NEP were mainly detected in the cytosolic fraction of the WT virus‐infected cells whereas were mainly found in the nuclear fraction of the mutant virus‐infected cells (Figure 1E). Consistently, the nuclear export protein CRM1 was significantly lower in cytoplasm while higher in the nucleus in the mutant viruses‐infected cells compared with the WT (Figure 1E). In addition to participating in vRNP nuclear export, NEP can also regulate influenza viral polymerase activity.^[^ ^16^ ^]^ We found overexpression of NEP S23C, S24L, or S25L does not significantly affect NEP's function in polymerase activity (Figure S1, Supporting Information). Collectively, these results suggest that the S‐S‐S motif in NEP plays a critical role in influenza vRNP nuclear export.

### 2.3 S23C, S24L, and S25L in NEP Interfere with NEP Association with CRM1

It has been proposed that NEP acts as an adaptor between the CRM1 and M1‐vRNP complexes. We wondered whether S‐S‐S motif affects vRNP nuclear export via modulating NEP interaction with M1 or CRM1. Co‐IP experiments were conducted to determine the effects of S23C, S24L, and S25L mutations on the NEP binding to CRM1 or M1. M1 was co‐expressed with NEP or NEP mutants in HEK293T cells and Co‐IP was performed using an anti‐NEP antibody. We found that all these NEP mutations significantly attenuated the ability of NEP to bind to CRM1 but barely affected the NEP‐M1 interaction (Figure 1G). To further confirm this in virus infection conditions, A549 cells were infected with H5N6 WT or NEP mutant viruses and Co‐IP was performed at 6 hpi. Consistently, NEP‐CRM1 interaction was significantly reduced by the S23C, S24L, and S25L mutations while the NEP‐M1 interaction was little influenced (Figure 1H). Taken together, S23C, S24L, and S25L in NEP interfere with NEP association with CRM1, further affecting the vRNP nuclear export.

### 2.4 S23A, S24A, and S25A Mutations in NEP Reduce Influenza Viral Replication

**Figure 2:**
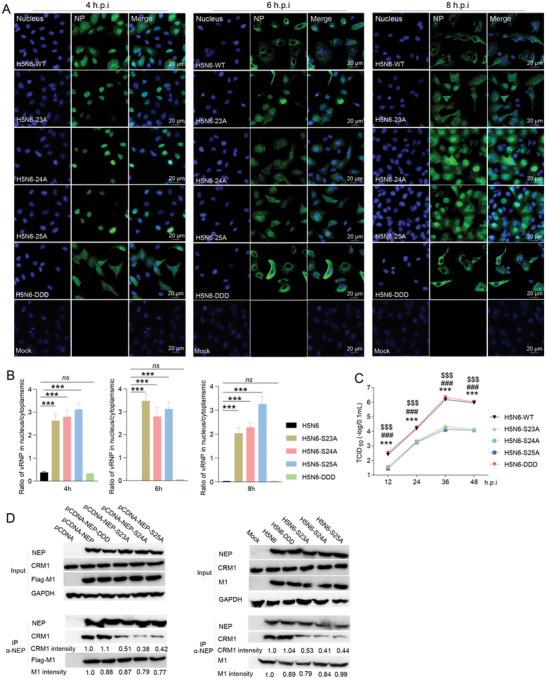
NEP S23A, S24A, and S25A mutations inhibit influenza A viral replication and vRNP nuclear export. A and B) The effect of S23A, S24A, S25A, and SSS/DDD mutations on influenza A vRNP nuclear export. A549 cells were infected with H5N6 wild‐type (WT) and the indicated NEP mutant virus, and NP distribution was detected at 4, 6, and 8 hours post‐infection (hpi.) (A). Representative images from three independent experiments were shown. Scale bar, 20 µm. The NP intensity in the nucleus and total NP intensity were quantified, and the ratio of vRNPs in the nucleus/cytoplasmic was shown B). Data are presented as mean ± SD (*n* = 6), ***p < 0.001, two‐tailed Student's *t*‐test. C) The effect of S23A, S24A, S25A and DDD mutations on influenza A virus replication. A549 cells were infected with H5N6 WT viruses or the NEP mutant virus at an MOI of 0.01 for 12, 24, 36, and 48 h. The viral titer was then measured by TCID_50_ assay. Data are presented as mean ± SD (*n* = 3). *** (S23A versus WT/DDD), ^###^ (S24A versus WT/DDD), and ^$$$^ (S25A versus WT/DDD), *p* < 0.001, two‐tailed Student's *t*‐test. D) The effects of S23A, S24A, S25A and DDD mutations on NEP‐CRM1 and NEP‐M1 interactions. HEK293T cells were co‐transfected with p3xflag‐M1 and pcDNA‐NEP or the mutants and were lysed at 24 h post‐transfection, and Co‐IP was performed using an anti‐NEP antibody. A549 cells were infected with H5N6 WT, NEP S23A, S24A, S25A or DDD mutant viruses for 6 h and Co‐IP was performed using an anti‐NEP antibody. CRM1 and M1 intensity were quantified.

To further confirm the important role of S‐S‐S motif in influenza virus replication, the de‐phosphorylation mutants of H5N6 NEP S23A, S24A and S25A as well as the constitutive phosphorylation NEP mutant S23D‐S24D‐S25D were created and the viruses harboring these NEP mutants were generated. The single NEP mutant S23A, S24A or S25A significantly inhibited the viral proliferation (**Figure** **2**C), and the triple mutant S23A‐S24A‐S25A viruses were failed to be rescued, suggesting that the S‐S‐S motif phosphorylation is required for influenza virus replication. However, the H5N6 virus containing the NEP S23D‐S24D‐S25D (H5N6‐DDD) did not promote the viral proliferation (Figure 2C), suggesting the constitutive phosphorylation of S‐S‐S motif may not benefit the virus replication. The effects of these mutant viruses on vRNP nuclear export and NEP‐CRM1 interaction were further determined. As expected, the de‐phosphorylation of S‐S‐S motif in NEP led to a weaker NEP‐CRM1 interaction and impaired vRNP nuclear export (Figure 2A,B,D). However, the constitutive phosphorylation of NEP S‐S‐S motif barely affected the NEP‐CRM1 interaction and vRNP nuclear export (Figure 2A,B,D). Thus, we conclude that the NEP S‐S‐S motif plays a critical role in influenza virus replication via regulating the vRNP nuclear export.

### 2.5 Inhibition or Deficiency of the Phosphorylation Kinase ATM and CK2 Impedes Influenza vRNP Nuclear Export and Viral Replication

**Figure 3:**
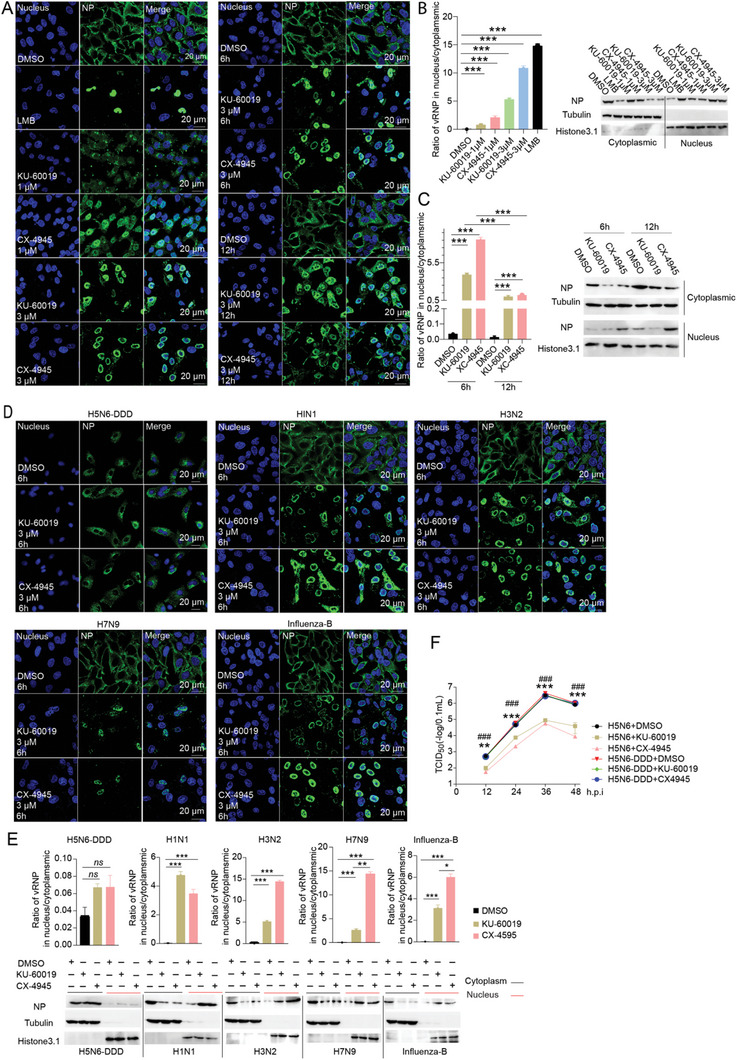
Inhibition of ATM and CK2 leads to delayed influenza vRNP nuclear export and reduced viral replication. A to C) The effect of different concentrations of ATM and CK2 inhibitors on influenza vRNP nuclear export. H5N6 virus‐infected A549 cells were treated with DMSO (negative control), 40 nM LMB (positive control), or 1 µM and 3 µM KU60019 (ATM inhibitor) and CX4945 (CK2 inhibitor) for 6 h. Representative images were shown for the NP staining. Scale bar, 20 µm. The ratio of vRNP distribution in the nucleus/cytoplasmic was quantified. Meanwhile, the cytoplasmic/nucleus extraction was performed followed by western blotting detection of NP distributions. Efficiency time of 3 µM of KU‐60019 and CX4945 on H5N6 vRNP nuclear export delay (A, right panel and C). Representative images were shown for the NP staining. Scale bar, 20 µm. The ratio of vRNP distribution in the nucleus/cytoplasmic is shown. D and E) Effects of KU‐60019 and CX4945 on the vRNP nuclear export of H5N6‐DDD, H1N1, H7N9, H3N2 and influenza B virus. A549 cells infected with the indicated viruses (MOI 0.01) were individually treated with the above inhibitors (3 µM), and NP distribution was detected at 6 hpi. Representative images from three independent experiments were shown. Scale bar, 20 µm. The ratio of vRNPs distribution in the cytoplasmic/nucleus was quantified. Meanwhile, the cytoplasmic/nucleus extraction was performed followed by western blotting detection of NP distributions. Data are presented as mean ± SD (*n* = 6). *p < 0.05; **p < 0.01, and ***p < 0.001, two‐tailed Student's *t*‐test. F) The effect of ATM and CK2 inhibitors on influenza A viral replication. A549 cells infected with H5N6 wild‐type or H5N6‐DDD viruses were treated with 3 µM of KU‐60019 or CX‐4945. The viral titers were measured at the indicated hpi. Data are presented as mean ± SD (*n* = 3). **p < 0.01; ***p < 0.001 (* CX‐4945 versus WT/ all the H5N6‐DDD groups; ^#^ KU‐60019 versus WT/ all the H5N6‐DDD groups), two‐tailed Student's *t*‐test.

To further determine the effect of NEP S‐S‐S motif phosphorylation on influenza virus replication, three strategies were utilized to block the phosphorylation of NEP S‐S‐S motif, and virus replication as well as vRNP nuclear export were examined. By using NetPhos 3.1 online service, two host Ser/Thr kinases ATM and CK2 were predicated as the candidates that potentially target S23, S24, or S25 in NEP. Next, we determined whether inhibiting ATM and CK2 activity could affect influenza vRNP nuclear export and virus replication. The cells were treated with ATM inhibitor KU‐60019, CK2 inhibitor CX‐4945, or CRM1 inhibitor leptomycin B (LMB) as a positive control, and vRNP nuclear export of H5N6 influenza virus was examined. KU‐60019 and CX‐4945 significantly inhibited vRNP nuclear export in a dose‐dependent manner (**Figure** **3**A,B), and the inhibition in vRNP nuclear export still can be observed after 12 hours of infection (Figure 3A,C). Interestingly, ATM and CK2 inhibitors have a broad effect on different types of influenza viruses, as demonstrated by that the vRNP nuclear export of different influenza A strains (H1N1, H3N2, and H7N9) and influenza B viruses were impaired by the inhibitors. However, the H5N6‐DDD mutant virus was little suppressed by both inhibitors (Figure 3D,E). In addition, the proliferation of influenza H5N6 virus was dramatically repressed under the treatment of above inhibitors while the H5N6‐DDD mutant virus was little affected (Figure 3F).

**Figure 4:**
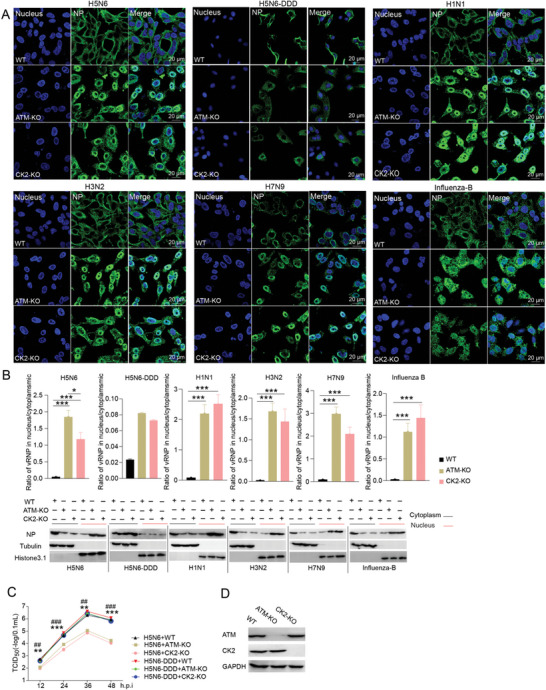
ATM or CK2 deficiency inhibits influenza vRNP nuclear export and viral replication. A) The vRNPs nuclear export in ATM‐ or CK2‐ deficient A549 cells. WT control and ATM or CK2 knockout A549 cells were infected with H5N6, H5N6‐DDD, H1N1, H3N2, H7N9 and influenza‐B virus at an MOI of 0.01, and vRNP nuclear export was examined at 6 hpi. Representative images were shown for NP staining. Scale bar, 20 µm. Quantification of the ratio of vRNP distribution in the nucleus/cytoplasmic and cytoplasmic/nucleus extraction result were shown in B). Data are presented as mean ± SD (*n* = 6). *p < 0.05 and ***p < 0.001, two‐tailed Student's *t*‐test. C) The proliferation of H5N6 wild‐type and H5N6‐DDD influenza viruses in WT and ATM or CK2 knockout A549 cells. The viral titers were measured by TCID_50_ assay. Data are presented as mean ± SD (*n* = 3). **p < 0.01 and ***p < 0.001, two‐tailed Student's *t*‐test. (*ATM‐KO versus WT/H5N6‐DDD groups; ^#^ CK2‐KO versus WT/ H5N6‐DDD groups). D) ATM and CK2 levels in A549 cells were detected by western blot. GAPDH was used as a loading control.

Meanwhile, influenza virus proliferation and vRNP nuclear export were examined in ATM‐ or CK2‐deficient cells. The ATM and CK2 knockout A549 cells were generated using the CRISPR/Cas9 technique, and the knockout efficiency of ATM and CK2 was confirmed by western blot (**Figure** **4**D). The vRNP nuclear export and viral replication were compared in WT and knockout cells. Similarly, the vRNP nuclear export of both influenza A, (including H5N6, H1N1, H3N2, H7N9) and influenza B viruses were significantly inhibited upon a loss of ATM or CK2 in cells, while the H5N6‐DDD mutant was little affected (Figure 4A,B). Correspondingly, the replication of H5N6 viruses was significantly reduced in both ATM and CK2 knockout cells compared to that in the WT cells while H5N6‐DDD mutant virus replicated similarly in these cells (Figure 4C). Taken together, either inhibition of ATM and CK2 activity or deficiency of ATM and CK2 impedes influenza vRNP nuclear export as well as viral replication of WT viruses but not the constitutive phosphorylation H5N6‐DDD mutant viruses, suggesting an important role of these two kinases in vRNP nuclear export and indicating a strong connection between the phosphorylation of NEP S‐S‐S motif and these two kinases.

### 2.6 NEP S‐S‐S Motif Mimics Inhibit Influenza vRNP Nuclear Export and Viral Replication

**Figure 5:**
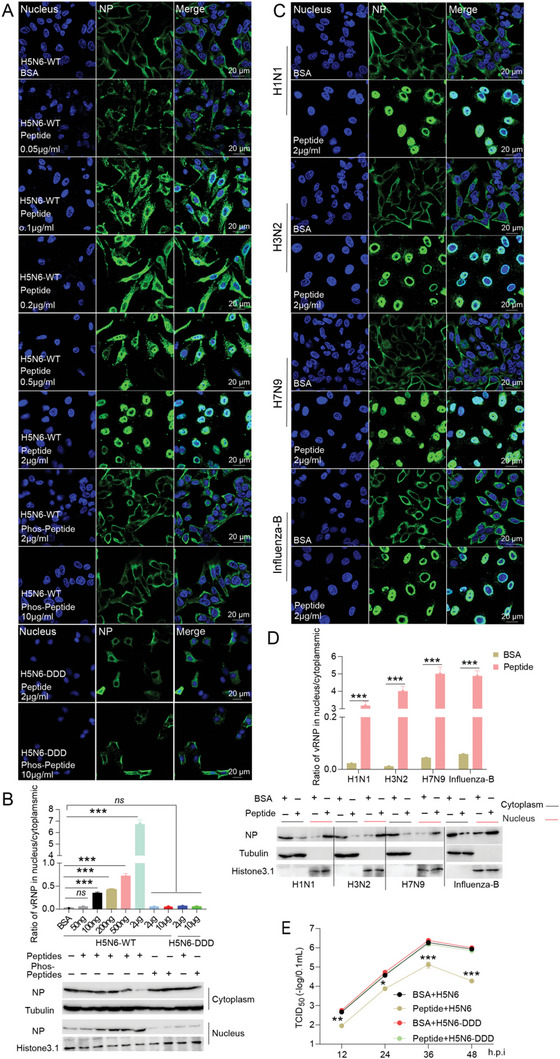
NEP S‐S‐S motif mimics inhibit influenza vRNP nuclear export and viral replication. A and B) The effects of different doses of NEP S‐S‐S motif peptide mimics (peptide) or phosphorylated peptide mimic (phos‐peptide) on influenza vRNP nuclear export. A549 cells infected with H5N6 or H5N6‐DDD influenza virus (MOI = 0.01) were treated with NEP peptide (0.05, 0.1, 0.2, 0.5, and 2.0 µg ml^−1^), phos‐peptide (2.0 and 10 µg ml^−1^), or BSA as a negative control, and vRNPs nuclear export was examined at 6 hpi. Representative images from three independent experiments were shown. Scale bar, 20 µm. The ratio of the vRNPs distribution in the cytoplasmic/nucleus and cytoplasmic/nucleus extraction results were shown in (B). C and D) A549 cells were infected with H1N1, H3N2, H7N9, and influenza‐B virus and were treated with 2.0 µg ml^−1^ of NEP peptide or BSA, and vRNPs nuclear export was examined at 6 hpi. The ratio of the vRNPs distribution in the nucleus/cytoplasmic and the cytoplasmic/nucleus extraction results were shown in (D). E) The effect of NEP S‐S‐S motif peptide mimics on influenza A virus replication. H5N6 or H5N6‐DDD influenza virus infected A549 cells were treated with NEP mimic peptide (2 µg ml^−1^) was measured by TCID_50_. Data are presented as mean ± SD (*n* = 3). *p < 0.05, **p < 0.01, and ***p < 0.001, two‐tailed Student's *t*‐test.

In the further study, a synthetic peptide containing NEP S‐S‐S motif was used to compete with NEP for binding to its phosphorylation kinases, and the vRNP nuclear export and virus replication were evaluated in the cells in which the phosphorylation is blocked by the peptide mimics. Intriguingly, the peptide mimics significantly inhibited the vRNP nuclear export of influenza H5N6 with a striking effect on it when using a concentration of 2 µg mL^−1^ (**Figure** **5**A,B), however, the peptide mimics in which the S23, S24, and S25 are phosphorylated (Phos‐Peptide) failed to do even at an extremely high concentration (Figure 5A,B). Again, both the NEP peptide mimics and the phosphorylated peptide had little effect on the vNRP nuclear export of H5N6‐DDD virus. Moreover, the NEP peptide mimics containing the single or double mutations of S23A, S24A, and S25A partly reversed the inhibition in vRNP nuclear export caused by NEP peptide mimics, while peptide mimics containing the triple S/A mutations eliminated the inhibition of NEP peptide mimics in vRNP nuclear export (Figure S2, Supporting Information). In addition, the vRNPs nuclear export of all other subtypes of influenza viruses tested in this study, including H1N1, H3N2, H7N9 and influenza B viruses, were inhibited by the NEP peptide mimics treatment (Figure 5C,D), indicating the NEP S‐S‐S motif mimics can extensively suppress different strains of influenza viruses. Finally, the effect of the NEP S‐S‐S motif mimic on influenza virus replication was examined and the data showed that it significantly inhibited influenza virus replication at a concentration of 2 µg mL^−1^ (Figure 5E). Collectively, blocking the phosphorylation of NEP S‐S‐S using the NEP peptide mimic impairs vRNP nuclear export and thus reduces the virus replication, further supporting an important role of phosphorylation on NEP S‐S‐S motif in influenza viral replication and vRNP nuclear export.

**Figure 6:**
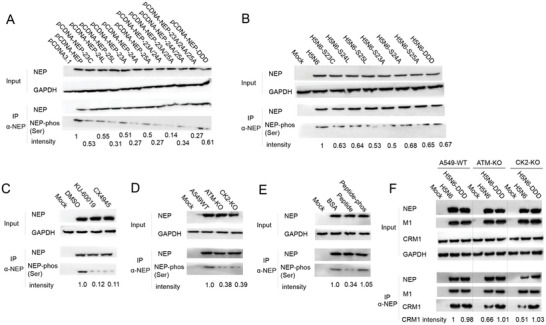
Detection of the total levels of serine phosphorylation when impairing phosphorylation of S‐S‐S motif. A) Detection of NEP mutants’ phosphorylation using a serine specific phosphorylation antibody in NEP‐overexpressed cells. 293T cells were transfected with NEP or its mutants for 24 h and lysed for IP experiments using the anti‐NEP antibody, followed by a western blot using an anti‐serine phosphorylation antibody. NEP serine phosphorylation levels were quantified using imageJ. B) Phosphorylation levels of NEP mutants in virus‐infected cells. A549 cells were infected with H5N6 wild‐type or the mutant viruses for 12 h and were lysed for western blotting to detect the NEP phosphorylation levels. C) The effect of inhibitors on NEP phosphorylation under viral infection. A549 cells were infected with H5N6 wild‐type virus followed by treating KU‐60019 or CX‐4945 inhibitors. NEP phosphorylation levels were detected at 12 h post infection. D) NEP phosphorylation levels in the wild‐type virus‐ infected ATM‐KO and CK2‐KO cells. E) Effects of NEP peptide mimics on the phosphorylation levels of NEP upon viral infection. A549 cells were infected with H5N6 wild‐type virus and treated with 2 µg mL^−1^ peptide mimics, or the phosphorylated peptides, and NEP phosphorylation levels were detected at 12 hpi. F) Detection of the NEP‐CRM1 interaction in H5N6 WT or DDD mutant virus‐infected cells and ATM/CK2 knockout cells. A549 WT or knockout cells were infected with H5N6 WT or DDD mutant viruses, respectively. Cells were lysed at 6 hpi followed by western blotting.

Loss of phosphorylation in NEP S‐S‐S motif reduces the total levels of NEP phosphorylation on serine residues. We have established an important role of NEP S‐S‐S motif phosphorylation in influenza replication, however, it is unclear whether NEP S‐S‐S motif could be indeed phosphorylated during influenza virus infection. Our attempt to develop an antibody specifically recognizing the phosphorylation of NEP S‐S‐S motif was failed. Instead, we measured the total serine phosphorylation levels on overexpressed‐NEP wild‐type or mutants. HEK293T cells were transfected with the WT NEP or NEP S23C, S24L, S25L mutants along with single, double and triple S/A mutants and the DDD mutant for 24 h, and the IP using an anti‐NEP antibody was performed followed by a western blot to detect the total levels of NEP serine phosphorylation. Our data show that the dephosphorylation S/A, S/C or S/L mutation significantly reduced the serine phosphorylation levels of NEP (**Figure** **6**A), indicating that the dephosphorylation mutations on NEP S‐S‐S motif indeed reduce the phosphorylation levels of NEP. The total levels of NEP serine phosphorylation were further examined in virus‐infected cells. We found that NEP serine phosphorylation levels were significantly decreased by S/C, S/L or S/A single mutation (Figure 6B), further supporting the phosphorylation of NEP S‐S‐S motif. Moreover, the NEP serine phosphorylation levels were detected in ATM and CK2 inhibitors‐treated cells and ATM or CK2 knockout cell lines as well as in mimic peptide treated cells. When treating with inhibitor KU‐60019 or CX‐4945, the phosphorylation level of NEP was significantly decreased (Figure 6C), which also confirmed in the ATM and CK2 knock‐out cell lines (Figure 6D). The mimic peptides similarly reduced the serine phosphorylation levels of NEP whereas the phosphorylated peptides barely affected that (Figure 6E). Collectively, the above results suggest that NEP S‐S‐S motif can undergo phosphorylation and the ATM and CK2 kinases likely be responsible for the NEP phosphorylation. To further confirm the role of ATM and CK2 in NEP S‐S‐S phosphorylation during viral infection, the CRM1‐NEP binding affinity were compared in WT and ATM‐ or CK2‐ deficient cells when infected with the H5N6 WT or H5N6‐DDD mutant viruses. Either ATM or CK2 deficiency significantly attenuated the NEP association with CRM1 in the H5N6 WT virus‐infected cells but not in the H5N6‐DDD mutant virus‐infected cells (Figure 6F), which emphasis the role of NEP phosphorylation in regulating NEP‐CRM1 interaction.

### 2.7 S23C, S24L, and S25L Mutations in NEP Result in Reduced Virulence of the Influenza Virus in Mice

**Figure 7:**
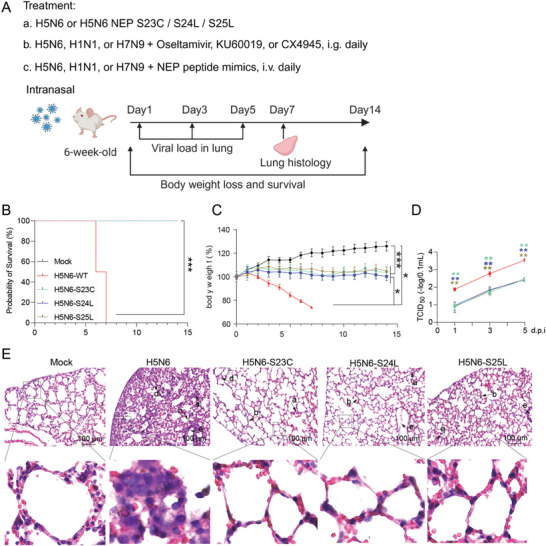
NEP S23C, S24L, and S25L mutations lead to reduced influenza virulence in mice. A) Schematic diagram showing the influenza virus challenge experiments in this study. H5N6 WT and NEP S23C, S24L, or S25L mutant virus challenge (a); KU‐60019 and CX‐4945 treatment (b) or NEP mimic peptide treatment (c) following H5N6, H1N1, and H7N9 influenza virus challenge. The diagram was created by BioRender online. B and C) Mice were infected with H5N6 WT or NEP mutant virus. Mouse survival and body weight were monitored daily for 14 days. Percentage of survival (B) and body weight (C) were analyzed. Data are presented as mean ± SD (*n* = 20 mice per group). *p < 0.05 and ***p < 0.001, two‐way ANOVA. D) Mice were sacrificed at 1, 3, and 5 dpi, and virus titers in the lung were measured by using TCID_50_ assays. Data are presented as mean ± SD (*n* = 4 mice per group). **p < 0.01, two‐tailed Student's *t*‐test. E) Mice were sacrificed at 7 dpi for lung histological analysis. Representative images from each condition were shown. Scale bar, 100 µm. The pathological changes in the lung include infiltration of inflammatory cells (a), alveolar wall thickening (b) alveolar wall capillary hyperemia (c), alveoli containing edema (d), and necrosis of bronchiole with cell derris in bronchiolar lumen (e).

We have shown that the S‐S‐S motif in NEP plays a critical role in influenza virus replication in vitro; we wondered whether it could affect the viral infection in mice. The effect of S23C, S24L, and S25L on influenza virus virulence in mice was examined. Six‐week‐old female BABL/c mice were infected intranasally (i.n.) with H5N6 WT or NEP S23C, S24L, or S25L mutant viruses at a dose of 2 x LD 50 (**Figure** **7**A‐a), and survival and body weight loss in these mice were monitored. WT virus‐infected mice showed significant weight loss starting at 2 days post‐infection (dpi), and all mice succumbed to infection by day 7 (Figure 7B,C). However, body weight was little changed in the mice infected with the mutant viruses and all these mice survived the infection (Figure 7B,C), which indicates that NEP mutant viruses are attenuated compared with WT viruses. The viral titers in the lungs of infected mice on days 1, 3, and 5 post‐infection were measured by TCID_50_ test_,_ and the results showed that the virus titers of the mutant viruses in the lung were significantly lower than the H5N6 WT virus (Figure 7D). Moreover, the histological analyses of the mouse lung were performed to examine the pathology caused by influenza virus infection. The mock control mice exhibited a normal phenotype with large air spaces, thin alveolar walls, and a clear alveolar structure in the lung (Figure 7E). Severe pathologies were observed in the WT virus‐infected mouse lung at 7 dpi, including serious infiltration of inflammation, alveolar wall thickening, alveoli containing edema, and necrosis of bronchiole with cell debris in the bronchiolar lumen. While the mice infected with NEP mutant viruses exhibited a mild pathology in the lung (Figure 7E). Taken together, these results from the mice suggest NEP S23C, S24L, and S25L largely attenuate influenza virus virulence.

### 2.8 Inhibition of ATM and CK2 in Mice Reduces Influenza Virus Infection

**Figure 8:**
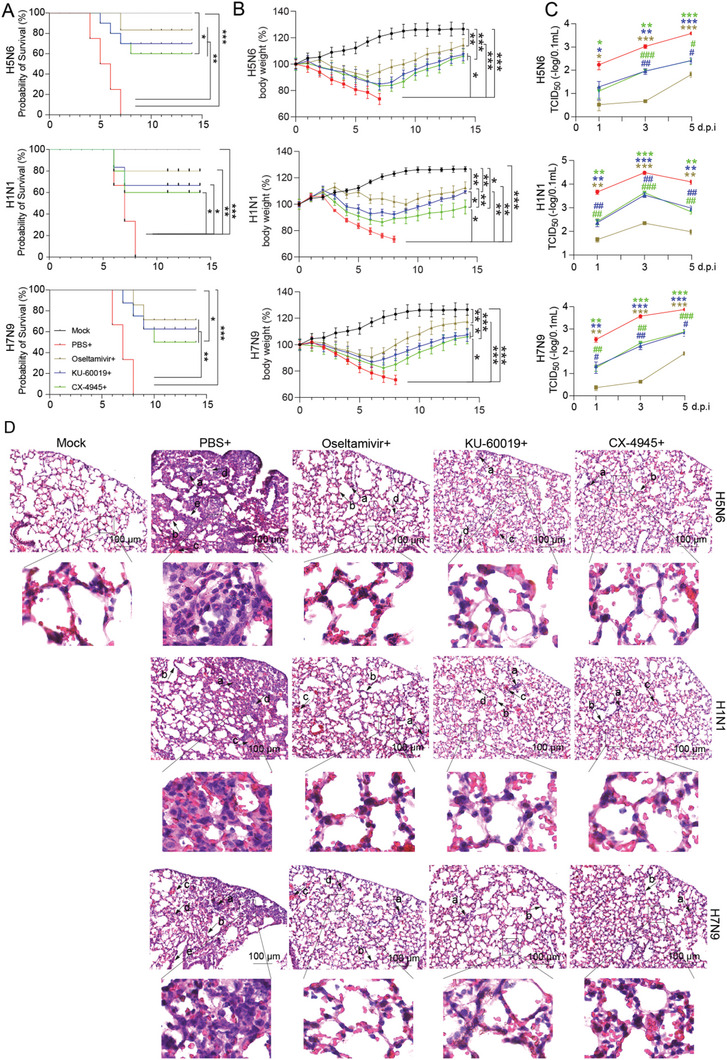
Inhibition of ATM and CK2 reduces influenza viral infection in mice. Mice were infected with the influenza virus and treated with ATM or CK2 inhibitor from 6 hours to day 5 post infection as indicated in Figure 5A‐b. A and B) Mouse survival rate (A) and body weight change (B) in the inhibitors‐treated mice infected with H5N6, H1N1, or H7N9 influenza viruses. Data are presented as mean ± SD (*n* = 20 mice per group). *p < 0.05, **p < 0.01, and ***p < 0.001, two‐way ANOVA. C) The viral titers in the lungs of the inhibitors‐treated mice infected with the indicated influenza virus. Data are presented as mean ± SD (*n* = 4 mice per group). *p < 0.05, **p < 0.01, and ***p < 0.001, two‐tailed Student's *t*‐test. (*compared with the PBS+ infected group; ^#^ compared with the Oseltamivir+ infected group). D) The pathological changes in the lungs of the inhibitors‐treated mice infected with the indicated influenza virus. The features of pulmonary pathology caused by influenza viral infection are described in Figure 5E. Representative images from each condition were shown. Scale bar, 100 µm.

To determine whether blocking NEP S‐S‐S motif phosphorylation could affect virus replication in vivo, the effect of ATM and CK2 inhibitors on viral infection was examined in mice. The ATM inhibitor KU‐60019, CK2 inhibitor CX‐4945, or the known anti‐viral drug Oseltamivir used as a positive control, were administrated daily in the influenza virus‐infected mice (Figure 7A‐b), and body weight and survival were monitored daily. Both KU‐60019 and CX‐4945 treatments reduced viral infection in mice, as demonstrated by significantly decreased mortality, less weight loss, and lower viral loads in the lung of the inhibitors‐treated mice compared with PBS‐treated control mice (**Figure** **8**A–C). In addition, KU‐60019 or CX‐4945‐treated mice developed modest pathology in the lung compared to the control mice with infiltration of inflammation, alveolar wall thickening, alveolar wall capillary hyperemia, alveoli containing edema, or cell debris in the lung (Figure 8D). Similarly, the body weight, survival, and viral titers in the lung were examined in the mice infected with H1N1 and H7N9, and we found that KU‐60019 and CX‐4945 can also reduce the infections of these viruses in mice (Figure 6A–C) and suggesting inhibition of the kinase ATM and CK2 leads to decreased virus replication in vivo.

### 2.9 Treatment of NEP S‐S‐S Motif Peptide Mimics Reduces Influenza Viral Infection in Mice

**Figure 9:**
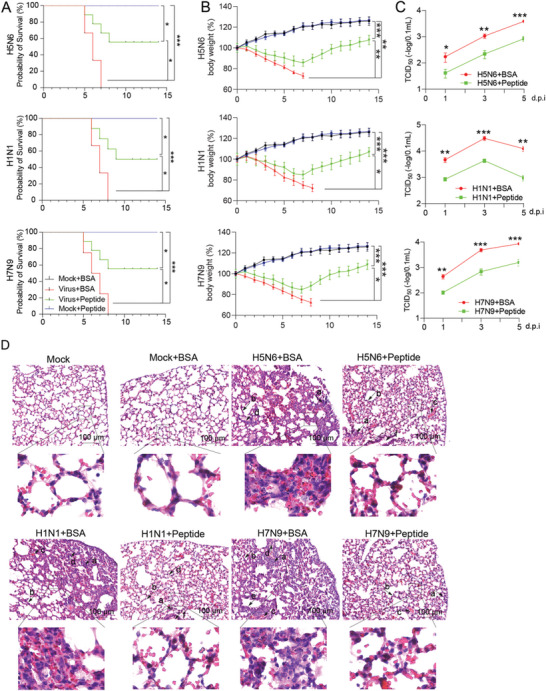
Treatment of NEP S‐S‐S motif mimics results in decreased influenza viral infection in mice. Mice were infected with influenza virus and treated with NEP S‐S‐S motif mimics as indicated in Figure 5A‐c. A and B) Mouse survival rate (A) and body weight change (B) in the NEP peptides‐treated mice infected with H5N6, H1N1, or H7N9 influenza virus. Data are presented as mean ± SD (*n* = 20 mice per group). *p < 0.05, **p < 0.01, and ***p < 0.001, two‐way ANOVA. C) The viral titers in the lungs of the peptides‐treated mice infected with the indicated influenza virus. Data are presented as mean ± SD (*n* = 4 mice per group**p < 0.01, and ***p < 0.001, two‐tailed Student's *t*‐test. D) The pathological changes in the lungs of the peptides‐treated mice infected with the indicated influenza virus. The features of pulmonary pathology caused by influenza viral infection are described in Figure 5E. Representative images from each condition were shown. Scale bar, 100 µm.

The effect of NEP S‐S‐S motif mimics on influenza virus infection in mice was also assessed. The mice infected with influenza viruses were treated with NEP peptide mimics, and the body weight, survival, viral titers in the lung, and pathology in the lung were examined (Figure 7A‐c). Interestingly, the peptide mimics treatment significantly reduced the mortality, weight loss, and viral load in the lung in the mice infected with H5N6, H1N1, or H7N9 influenza viruses (**Figure** **9**A–C). As shown in the H&E staining of the lung samples from the virus‐infected mice, the NEP peptide mimics treatment significantly alleviated the pathologies caused by the infections of H5N6, H1N1, and H7N9 influenza viruses (Figure 9D). These results suggest that NEP S‐S‐S motif mimics treatment reduces influenza viral infection in mice. In summary, the above results from the mouse studies provide evidence supporting an important role of the NEP S‐S‐S motif phosphorylation in influenza virus replication in vivo.

## 3 Discussion

NEP plays a crucial role in the influenza viral life cycle mainly through regulating the nuclear export of vRNPs.^[^ ^4^, ^17^, ^18^ ^]^ However, the detailed mechanism how NEP regulates vRNP nuclear export remains fully dissected. In this study, we demonstrate a critical role of the NEP S‐S‐S motif in the nuclear export of vRNPs by regulating NEP interaction with the cellular nuclear export factor CRM1. Importantly, we provide evidence that targeting the NEP S‐S‐S motif has the potential to develop novel antiviral strategies against both influenza A and B viruses.

### 3.1 Role of NEP S‐S‐S Motif in Influenza Viral Replication

One central function of NEP in virus replication is regulating the vRNP nuclear export which is essential for influenza virus replication.^[^ ^4^ ^]^ A highly conserved S‐S‐S motif has been identified in NEP of influenza A viruses,^[^ ^13^, ^15^ ^]^ which has been speculated that it might affect NEP activity in vRNP nuclear export or polymerase activity‐enhancing function. To explore the role of NEP S‐S‐S motif in influenza virus replication, we generated the NEP mutant virus in which the phosphorylation on NEP S‐S‐S is defective. Considering that substitution of NEP S23, 24, and 25 either to A or to D will lead to mutations in amino acids of NS1 since they are translated from an alternatively spliced RNA derived from the same genome segment,^[^ ^19^ ^]^ we mutated S23, S24, and S25 to cysteine (C), leucine (L), and L, respectively. We found that S23C, S24L, or S25L leads to drastic decreases in virus replication in cells as well as reduced virus virulence in mice (Figures 1, 7). In addition, it appears that each of these serine residues contributes equally to the virus replication and the efficient viral replication requires at least two of them because single mutation similarly inhibits virus replication and simultaneously mutating two or three of them causes failed virus rescue (Figure 1B,F). These observations indicate a critical role of the NEP S‐S‐S motif in influenza virus replication. Going further, we show that S23C, S24L, or S25L impedes the vRNP nuclear export but does not affect polymerase activity compared to the WT NEP (Figure 1C–E; Figure S1, Supporting Information). However, a previous study demonstrated that mutation of S23, S24, and S25 to either alanine (A, dephosphorylation) or aspartic acid (D, mimicking phosphorylation) barely impacts replication of influenza A virus,^[^ ^14^ ^]^ which is inconsistent with our findings. While the discrepancy in these results between Reuther et al and ours remains obscure. To further clarify the importance of NEP S‐S‐S motif phosphorylation in influenza virus replication, the effect of dephosphorylation (S23A, S24A, and S25A) and mimicking phosphorylation (S23D‐S24D‐S25D) NEP mutations on virus replication were detected as well. We found S to A single mutations have similar effect on virus replication as the S23C, S24L, and S25L mutations and D‐D‐D mutations do not impact the virus replication (Figure 2). Therefore, we would like to emphasize here that the S‐S‐S motif of NEP is important for virus replication. In addition to that mutating one of the serine sites in S‐S‐S motif dramatically reduces virus replication in *vitro* and in *vivo* (Figures 1, 7), blocking the phosphorylation by either inhibiting the putative kinase ATM and CK2 for S‐S‐S motif or competing binding to its kinases using NEP S‐S‐S motif mimics results in impaired vRNP nuclear export and reduced virus replication, which indicates a potential role of the phosphorylation on NEP S‐S‐S motif in influenza virus replication. However, due to the lack of antibodies that specifically detect the phosphorylated S‐S‐S motif of NEP, we are only able to analyze the changes of total NEP serine phosphorylation levels under the conditions that NEP S‐S‐S motif phosphorylation is supposed to be impaired by dephosphorylation mutations, CK2 and ATM2 inhibition or deficiency, or NEP peptides mimics treatment. Our data suggests that NEP S‐S‐S motif phosphorylation is present in viral infection and is important for virus replication. First, the total serine phosphorylation levels of NEP were significantly decreased by de‐phosphorylation mutation in S‐S‐S motif in both overexpression and infection conditions (Figure 6A,B), indicating the loss of phosphorylation mutation on NEP S‐S‐S motif indeed impairs NEP serine phosphorylation. Secondly, ATM or CK2 inhibition and deficiency as well as NEP peptide mimics treatment significantly reduced the NEP serine phosphorylation when infected with H5N6 wild‐type virus (Figure 6C,D), suggesting ATM or CK2 may mediate the phosphorylation of NEP S‐S‐S motif (Figure 6E). Last, the NEP‐CRM1 interaction was reduced in H5N6 wild‐type virus infection but little affected in the H5N6‐DDD mutant virus infection in the ATM or CK2 knockout cells (Figure 6F), which not only demonstrates that S‐S‐S motif phosphorylation regulates NEP‐CRM1 interaction thereby affecting vRNP nuclear export and viral replication but also suggests a role of ATM and CK2 in NEP S‐S‐S phosphorylation. Although in vitro kinase assay is required to further determine whether CK2 and ATM could directly phosphorylate NEP S‐S‐S motif, our study provides evidences that phosphorylation of NEP S‐S‐S motif, likely mediated by the host kinase CK2 and ATM, plays a critical role in influenza virus replication.

### 3.2 Regulation of the NEP S‐S‐S Motif in Influenza vRNP Nuclear Export

It was established that NEP mediates nuclear export by interacting with the cellular nuclear export protein CRM1 and the viral M1 protein, which is in turn bound to vRNPs.^[^ ^1^, ^4^, ^18^, ^20^ ^]^ However, a detailed mechanism of how NEP interacts with CRM1 to mediate the vRNP nuclear export is required for further determination. Previous studies have shown that the nuclear export signal (NES) in NEP is involved in NEP‐CRM1 interaction,^[^ ^11^ ^]^ while the contribution of each NES to NEP‐CRM1 interaction needs to be clarified. Although NES1 (amino acid 12–21) was initially found to be important for the vRNPs nuclear export and the NTD of NEP (amino acids 1–54) mediates NEP binding to the CRM1, the NES1 located in NTD is not required for the binding.^[^ ^21^, ^22^ ^]^ In addition, several studies have shown that the NEP‐CRM1 interaction is enhanced when deleting NES1 while is abolished when deleting both NES1 and NES2 (amino acids 31–42).^[^ ^9^ ^]^ These observations indicate that NES1 and NES2 may cooperate to regulate the interaction between NEP and CRM1 during influenza viral infection. Herein, we show that mutating S‐S‐S motif in NEP impairs the NEP interaction with CRM1 but not with M1 (Figure 1G,H). A possible mechanism for this is suggested by the cellular MK2 protein, requiring phosphorylation for CRM1‐mediated export as well. In MK2, the NES is part of an auto‐inhibitory alpha helix that interacts with an adjacent domain of the molecule. Phosphorylation of a hinge region induces the conformational change, which reduces the interaction between the alpha helix and the rest of the molecule and unmasks the NES.^[^ ^13^, ^23^, ^24^ ^]^ Similarly, the prediction of NEP‐NTD structure has shown that NES1 and NES2 reside in the alpha helix N1 and helix N2, respectively, and the S‐S‐S motif is located in the loop between these two helixes,^[^ ^25^ ^]^ the phosphorylation of which might modulate NEP conformation and thereby regulating the NEP‐CRM1 interaction. Additionally, the prediction of the structure of the NEP‐CRM1 complex using AlphaFold^[^ ^26^ ^]^ suggested that S23C, S24L, or S25L mutation on NEP may weaken the interaction between NEP and CRM1 (data not shown), hence, we cannot rule out that mutating the S to C or L may induce the NEP conformational change, thereby affecting the interaction between NEP and CRM1. Moreover, the CHX chase assay data showed that NEP S/C, S/L, S/A single, double or triple mutations little affected the stability of NEP protein; while the DDD mutation seem delayed the degradation of NEP protein in our condition which may related to the site‐specific (S/D) effect of phosphorylation on protein turnover^[^ ^27^ ^]^ (Figure S3, Supporting Information). These data indicate that the effect of mutations on virus replication don't caused by the alterations of protein stability. Although future studies are needed to further elucidate the mechanism, our research suggests that, in addition to NES1 and NES2 in NEP, the S‐S‐S motif that located in proximity to the NES likely plays an indispensable role in mediating NEP‐CRM1 interaction.

### 3.3 Insights into the Development of Anti‐Viral Targets or Strategies for Influenza A and B Viruses

Influenza virus is an important public health pathogen that causes considerable human morbidity and mortality every year.^[^ ^28^, ^29^ ^]^ While the development of vaccines and antivirals to prevent infection and treat infected people is feasible, it remains a significant challenge for public health due to the rapid mutation and recombination of influenza viruses.^[^ ^28^, ^30^ ^]^ The emergence of novel and/or drug‐resistant IAV strains fails available vaccines and antivirals. Therefore, novel vaccines and antiviral development are required to combat the threats posed by the influenza virus.

In this study, we provide novel insights into developing influenza vaccines or anti‐viral drugs. First, our studies suggest that modifying the NEP S‐S‐S motif could be an effective strategy for attenuating the virus, thereby generating a live‐attenuated vaccine to protect against the influenza. S‐S‐S motif is well conserved among influenza NEP proteins and NEP S23C, S24L, or S25L leads to significantly decreased virulence of influenza A virus in mice (Figures 1, 7), enabling the generation of an attenuated influenza vaccine strain. However, to further demonstrate the potential of NEP S‐S‐S motif mutant viruses as a live‐attenuated vaccine, investigation is required to assess the immunogenicity of the NEP S23C, S24L, or S25L mutant virus in a mouse model. Secondly, the host kinases ATM and CK2 might serve as potential targets for developing anti‐viral drugs for influenza. We found that the ATM inhibitor KU‐60019 and CK2 inhibitor CX‐4945 significantly inhibit influenza A and B viral replication in both cells and mice, albeit slightly less effectively than the well‐known antiviral drug Oseltamivir (Figures 3, 8). Consistently, previous studies have shown a role of CK2 in influenza virus replication by affecting virus budding or regulating NS1 function.^[^ ^31^, ^32^ ^]^ Here, we demonstrate that CK2 inhibition impairs the vRNP nuclear export (Figures 3, 4), revealing a novel role of CK2 in influenza virus replication and suggesting the possibility of targeting the host kinase as antivirals. Thirdly, NEP S‐S‐S motif peptide mimics might be used as anti‐viral peptide drugs against influenza viral infection. The peptide‐based therapeutics against viruses have emerged as a new field in antivirals development over the last few years, which have been approved for multiple viruses, including influenza virus.^[^ ^33^, ^34^, ^35^ ^]^ We show that the treatment of a NEP S‐S‐S motif peptide mimic, designed to competitively inhibit NEP binding to its kinases, reduces influenza viral infection in mice (Figure 9), indicating the potential of the NEP peptide mimic emerges as a novel antiviral drug candidate for influenza virus. In summary, our studies suggest that the phosphorylation of NEP S‐S‐S motif is required for the efficient replication of influenza virus, and targeting the NEP S‐S‐S motif might contribute to the development of novel influenza vaccines or antiviral drugs.

## 4 Experimental Section

### Ethics Statements

All animal studies in this manuscript were conducted following approval from Hubei Administrative Committee for Laboratory Animals (Approval No.: SYXK‐2015‐0084) and the Scientific Ethics Committee of Huazhong Agricultural University (Approval No.: HZAUMO‐2019‐018), in full compliance with the Care and Use of Laboratory Animals of the Ministry of Science and Technology of the People's Republic of China policies. All the infection experiments were performed in the biosafety level‐3 (BSL3) facility of Huazhong Agricultural University in accordance with the institutional biosafety manual. The animals were housed in negative pressure isolators with high‐efficiency particulate air filters in the BSL3 facility.

### Cells, Viruses, and Plasmids

HEK293T, MDCK, and A549 cells were maintained in DMEM (Gibco, NY, USA) or F12 (Hyclone, Beijing, China) medium supplemented with 10% fetal bovine serum (FBS), respectively, and were cultured at 37 °C under 5% CO_2_. Influenza A virus H5N6 and H3N2 were isolated and stored by our laboratory; H1N1 (PR8), H7N9, and H5N6 NEP S23C, S24L, S25L, S23A, S24A, S25A and NEP‐DDD mutant viruses were generated by reverse genetics using the pHW2000 plasmid system. Influenza B virus (Brisbane) was kindly gifted by Dr. Ze Chen (Shanghai Institute of Biological Products Co., LTD). H5N6 NEP and NEP mutations were cloned into the pCDNA3.1 vector (pCDNA‐NEP, pCDNA‐NEP‐S23C, S24L, S25L, S23A, S24A, S25A, 23A/24A, 23A/25A, 24A/25A, 23A/24A/25A and DDD). M1 was cloned into p3XFlag vector (Flag‐M1).

### Generation of ATM or CK2 Knockout Cell Line

The ATM and CK2 knockout cell lines were generated by using the CRISPR/Cas9 system.^[^ ^36^, ^37^ ^]^ The guider RNA sequences were designed online^[^ ^38^ ^]^ as follows: ATM: Forward, 5′‐TAGGagcagcgccaatatgatgtc‐3′, Reverse, 5′‐AAACgacatcatattggcgctgct‐3′; CK2: Forward, 5′‐TAGGagcagcgccaatatgatgtc‐3′, Reverse, 5′‐AAACgacatcatattggcgctgct‐3′. A549 cells cultured in 6‐well plates were transfected with px300‐ATM‐GFP or px300‐CK2‐GFP plasmid, respectively, followed by sorting the GFP‐positive cells using a flow cytometer at 48 hours post‐transfection. The GFP‐positive cells were collected and cultured for further experiments. Finally, the knockout efficiency was confirmed by western blot analysis.

### Co‐Immunoprecipitation (Co‐IP)

HEK293T cells were co‐transfected with p3xflag‐M1 and pCDNA‐NEP or its mutants S23C, S24L, and S25L and were lysed with RIPA buffer (Beyotime, China) after 24 hours, followed by co‐immunoprecipitation using the NEP polyclonal antibody and western blot analysis. To detect the interaction in virus‐infected cells, A549 cells were infected with H5N6 WT, S23C, S24L, or S25L with an MOI of 10, and cells were lysed at 6 hpi, followed by Co‐IP and western blot analyses as described above.

### Immunofluorescence Assay (IFA) and Confocal Microscope

To examine the vRNP nuclear export in A549 cells, cells on coverslips were infected with the indicated influenza viruses. The cells were fixed at 4, 6, 8, or 12 hpi, permeabilized with 0.1% TritonX‐100 solution, and blocked with 1% BSA for 1 h. The cells were then incubated with a homemade anti‐NP mouse monoclonal antibody, followed by incubation with the donkey anti‐mouse Alexa Fluor 488‐conjugated secondary antibody. Images were acquired using a confocal microscope (63 x objectives). To quantify the percentage of the vRNPs in the nucleus, at least 20 images in a random field view from each coverslip were quantified.

### Minireplicon Assay

Polymerase activity was measured by a minireplicon assay. In brief, HEK293T cells were cotransfected with pPolI‐luc, a Renilla luciferase expression plasmid, and four RNP expression plasmids, pCDNA3.1‐PB1, pCDNA3.1‐PB2, pCDNA3.1‐PA, and pCDNA3.1‐NP. Luciferase activity was measured using a Dual‐Luciferase assay kit (Promega) according to the manufacturer's protocol at 24 h post transfection. Renilla luciferase activity was used as an internal control to normalize transfection efficiency.

### TCID_50_ Assay

The A549 wild‐type, ATM, or CK2 knockout cells were infected with H5N6 WT or NEP‐ S23C, S24L, or S25L variant viruses with an MOI of 0.01. The supernatants were harvested at 12, 24, 36, and 48 hpi followed by the TCID_50_ assay. To measure the influenza virus titers in cell or mouse lungs, MDCK cells were cultured in the 96‐well plates before viral infection. Viral supernatants harvested from the cells or mouse lungs were serially diluted with DMEM and added to each well with eight replicates of each dilution. The cells were examined under the microscope at 72 hpi to identify cytopathic effects (CPE).^[^ ^39^, ^40^ ^]^ The 50% tissue culture infective dose (TCID50) was calculated using the Reed‐Muench method.

### Viral Infection in Mice, Administration of Inhibitors or Peptides in Mice, Sample Collection, and Lung Histological Analysis

Six‐week‐old female BABL/c mice were intranasally inoculated (i.n.) with 50 µL of 4 MLD_50_ (50% mouse lethal dose) of H5N6 (A/Chicken/Jiangxi/02/2016, 1.5×10^3^ TCID_50_ s mL^−1^), H5N6 variant viruses (1.5×10^3^ TCID_50_ s mL^−1^), H1N1 (A/Puerto Rico/8‐SV14/1934, 38 TCID_50_ s mL^−1^) or H7N9 (A/Shanghai/Mix1/2014, 1.6×10^4^ TCID_50_ s mL^−1^) influenza viruses, respectively. The body weight and survival were monitored daily for 14 days, and the body weight loss over 20% to 25% compared to the initial weight was subjected to morality and will be euthanasia. Oseltamivir (50 mg k^−1^g day^−1^), KU‐60019 (10 mg k^−1^g day^−1^, S1570, Selleckchem, Houston, TX, 77 054, USA), and CX‐4945 (75 mg k^−1^g day^−1^, S2248, Selleckchem, Houston, TX, 77 054, USA) were administrated daily via oral gavage (i.g.) from 6 hours to day 5 post‐infection; NEP S‐S‐S motif peptide mimic MQLASSSEDL or phos‐peptide MQLAS_p_S_p_S_p_EDL (4 mg k^−1^g day^−1^) were administrated daily by intravenous injection (i.v.) from 6 hours to day 5 post‐infection Mice were euthanasia by CO_2_ and mouse lung was collected at 1, 3, and 5 dpi. For virus burden detection, the lung was homogenized, and the supernatants were applied for TCID_50_ measurement. On day 7, three mice from each group were euthanasia. The lungs were collected and fixed in 4% PFA for 24 h followed by the paraffin sectioning and H&E staining according to a standard protocol.^[^ ^41^ ^]^ Twenty images were captured from each lung section using an Olympus microscope (BX53 system, 20 x objectives).

### Cycloheximide (CHX) Chase Assay

The cycloheximide (APExBIO, A8244, USA) chase assay was performed as described previously.^[^ ^42^ ^]^ HEK293T cells in 12‐well plate were transfected with NEP or NEP mutants (1 µg well^−1^) for 24 hours and the cycloheximide was added into the cells (20 µg mL^−1^) followed by lysing the cells at 0.5, 1, 1.5, and 2 hours after treatment and detection of NEP expression levels using western blot. Three independent experiments were performed and the NEP intensity was analyzed by image J. Half‐life curves of the WT and mutant NEP proteins were generated by normalizing the protein levels in the CHX‐treated cells to the levels in the untreated control cells.

### Statistical Analysis

All statistical analyses were performed using GraphPad Prism 9. Data were presented as mean ± SD. Statistical significance was assessed by unpaired two‐tailed Student's *t*‐test, one‐way ANOVA, or two‐ way ANOVA tests (for the body weight and survival analysis). P values less than or equal to 0.05 were considered statistically significant. *p < 0.05; **p < 0.01; ***p < 0.001.

## Conflict of Interest

The authors declare no conflict of interest.

## Authors’ Contributions

X.L. and C.Y. contribute equally to this work. X.L. performed the experiments, analyzed the data, and drafted the manuscript. C.Y. ran the NEP‐CRM1 structure prediction, analyzed the data, and revised the manuscript. X.L. prepared the plasmids. X.S., H.C., Q.Z., and M.J. analyzed the data. Q.Z. and M.J. support this study. M.J. supervised the project.

## Supporting information



Supporting Information

## Data Availability

The data that support the findings of this study are available from the corresponding author upon reasonable request.
