# Supplementary material for: Phosphorylation of S‐S‐S Motif in Nuclear Export Protein (NEP) Plays a Critical Role in Viral Ribonucleoprotein (vRNP) Nuclear Export of Influenza A and B Viruses
Source: Adv Sci (Weinh). 2024 Nov 22;12(2):2309477. doi: 10.1002/advs.202309477 (PMC11727112; doi:10.1002/advs.202309477)

**Fig.S1: Effects of NEP S23C, S24L, and S25L on influenza A polymerase activity.** HEK293T cells were transfected with NEP WT or mutant plasmids as indicated (0.05, 0.1, and 0.5 μg/ well) and viral RNP reconstitution plasmids (pCDNA-3.1-PB1, -PB2, -PA, and -NP, pPolI-Luc, and Renilla); polymerase activity was measured at 24 h post-transfection. Data are presented as mean ± SD (n=3), *, P<0.05, two-tailed Student’s t-test.

**Fig.S2 Effect of NEP mutant mimics peptide on virus vRNP nuclear export.** (A) A549 cell were infected with H5N6 wild-type virus and treating with 2μg/mL NEP mimics peptide or its mutants (including single, double or triple S/A mutations on S-S-S motif), and IFA was performed at 6 hours post infection. The ratio of vRNP distribution in nucleus/cytoplasmic were calculated after quantifications, Data are presented as mean ± SD (n=6), *, P<0.05, **, P<0.01, ***, P<0.001, two-tailed Student’s t-test.

**Fig.S3 Effects of mutations on NEP protein turnover. (A)** The expression levels of NEP WT and mutants. **(B)** The levels of NEP WT and mutants in HEK293T cells under CHX treatment. HEK293T cells were transfected with WT NEP or its mutants for 24 h. Cells were treated with CHX for 0.5, 1, 1.5, and 2 h. The NEP and mutant levels were quantified and their degradation curves were generated from three independent experiments. Data are presented as mean ± SD (n=3), *, P<0.05, **, P<0.01, ***, P<0.001, by two- way ANOVA tests.

Fig.S1


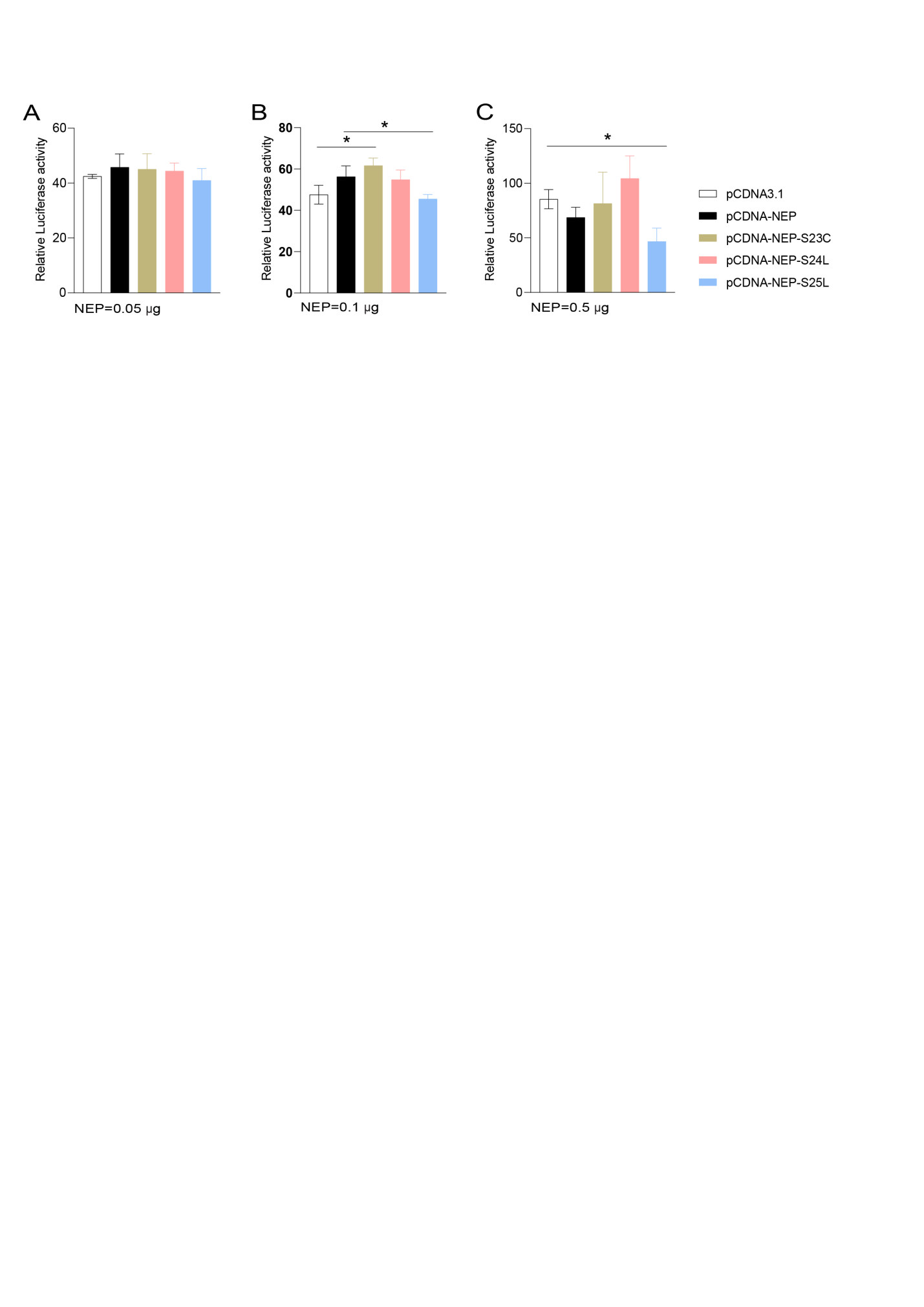


Fig.S2


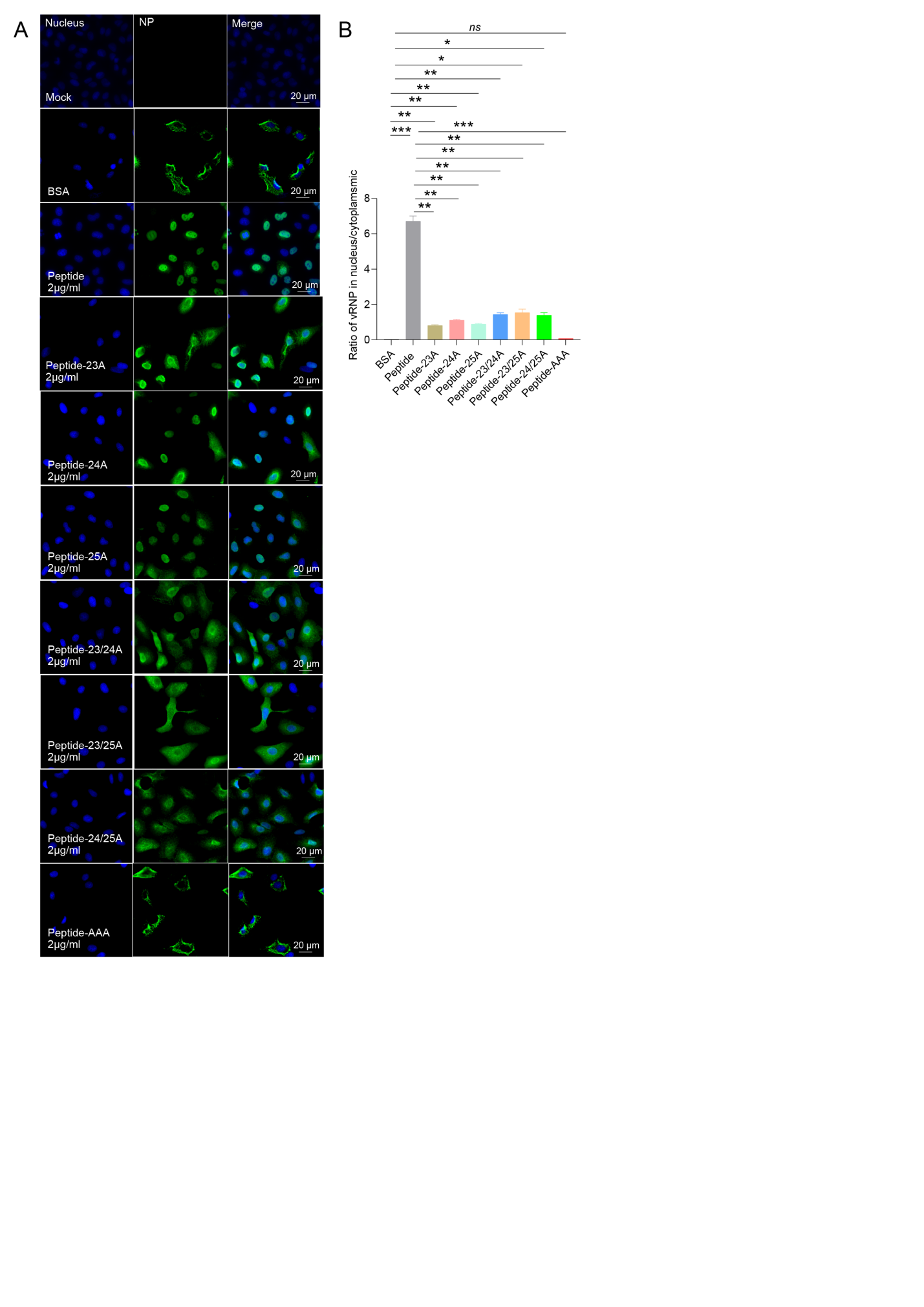


**Fig.S3**


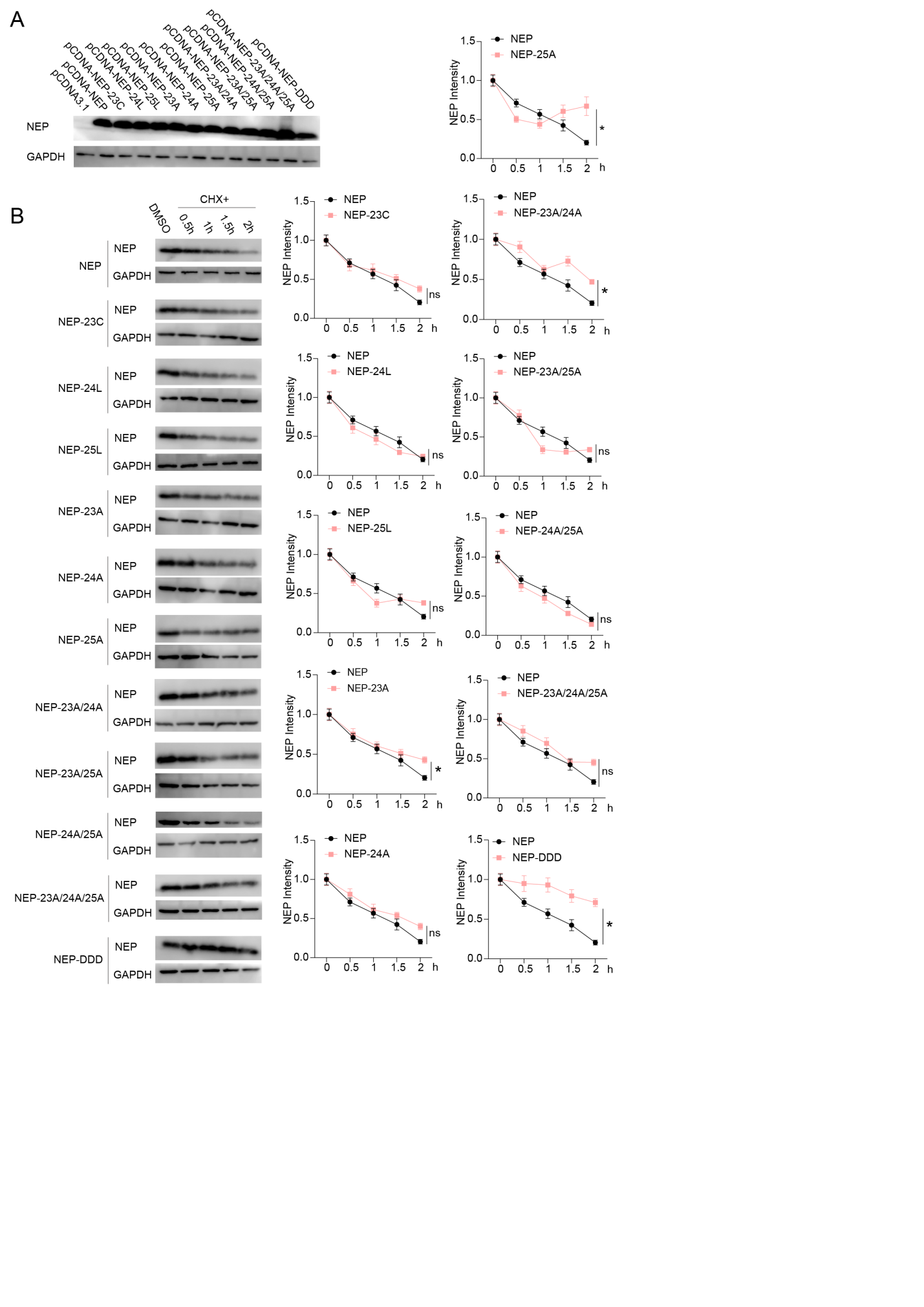

Supplement: Supplementary file 1 — Supporting Information [file ADVS-12-2309477-s001.docx]
